# A DLG2 deficiency in mice leads to reduced sociability and increased repetitive behavior accompanied by aberrant synaptic transmission in the dorsal striatum

**DOI:** 10.1186/s13229-020-00324-7

**Published:** 2020-03-12

**Authors:** Taesun Yoo, Sun-Gyun Kim, Soo Hyun Yang, Hyun Kim, Eunjoon Kim, Soo Young Kim

**Affiliations:** 1 Department of Biological Sciences, Korea Advanced Institute for Science and Technology (KAIST), Daejeon, 34141 Korea; 2 Center for Synaptic Brain Dysfunctions, Institute for Basic Science (IBS), Daejeon, 34141 Korea; 3 Department of Anatomy, College of Medicine, Korea University, Seoul, 02841 Korea; 4 College of Pharmacy, Yeungnam University, Gyeongsan, 38541 Korea

**Keywords:** Autism, PSD-93, Locomotion, Social interaction, Self-grooming, Striatum, Spiny projection Neurons

## Abstract

**Background:**

DLG2, also known as postsynaptic density protein-93 (PSD-93) or chapsyn-110, is an excitatory postsynaptic scaffolding protein that interacts with synaptic surface receptors and signaling molecules. A recent study has demonstrated that mutations in the DLG2 promoter region are significantly associated with autism spectrum disorder (ASD). Although DLG2 is well known as a schizophrenia-susceptibility gene, the mechanisms that link DLG2 gene disruption with ASD-like behaviors remain unclear.

**Methods:**

Mice lacking exon 14 of the *Dlg2* gene (*Dlg2*^*–/–*^ mice) were used to investigate whether *Dlg2* deletion leads to ASD-like behavioral abnormalities. To this end, we performed a battery of behavioral tests assessing locomotion, anxiety, sociability, and repetitive behaviors. In situ hybridization was performed to determine expression levels of *Dlg2* mRNA in different mouse brain regions during embryonic and postnatal brain development. We also measured excitatory and inhibitory synaptic currents to determine the impacts of *Dlg2* deletion on synaptic transmission in the dorsolateral striatum.

**Results:**

*Dlg2*^*–/–*^ mice showed hypoactivity in a novel environment. They also exhibited decreased social approach, but normal social novelty recognition, compared with wild-type animals. In addition, *Dlg2*^*–/–*^ mice displayed strong self-grooming, both in home cages and novel environments. *Dlg2* mRNA levels in the striatum were heightened until postnatal day 7 in mice, implying potential roles of DLG2 in the development of striatal connectivity. In addition, the frequency of excitatory, but not inhibitory, spontaneous postsynaptic currents in the *Dlg2*^*–/–*^ dorsolateral striatum was significantly reduced.

**Conclusion:**

These results suggest that homozygous *Dlg2* deletion in mice leads to ASD-like behavioral phenotypes, including social deficits and increased repetitive behaviors, as well as reductions in excitatory synaptic input onto dorsolateral spiny projection neurons, implying that the dorsal striatum is one of the brain regions vulnerable to the developmental dysregulation of DLG2.

## Background

DLG2, also known as postsynaptic density protein-93 (PSD-93) or Chapsyn-110, is a postsynaptic scaffold protein that belongs to the membrane-associated guanylate kinase (MAGUK) family, whose members directly interact with diverse synaptic receptors, membrane proteins, and signaling proteins and play a critical role in the molecular organization of multi-protein complexes in the postsynaptic density at excitatory synapses [1–5]. DLG2 protein is abundantly expressed throughout the adult rodent brain, including in the cortex, hippocampus, striatum, and cerebellum [2, 3].

Despite its prominent expression in the cerebellum, *Dlg2* deletion in mice does not alter the development or synaptic function of parallel fibers in the cerebellum or motor coordination [6]. On the other hand, a DLG2 deficiency in the hippocampus leads to reduced synaptic long-term potentiation (LTP) [7], which contrasts with the effects of a deficiency of DLG4 (also known as PSD-95) that enhances LTP in the mouse hippocampus [7, 8]. These previous findings suggest that the roles of DLG2 in synaptic connectivity may vary in different brain regions.

It has been established that genetic variations in *DLG2* are associated with neurodevelopmental disorders, including schizophrenia [9–13] and intellectual disability [14]. For example, de novo loss-of-function mutations in *DLG2* have been repeatedly found in schizophrenia patients [11, 13]. In addition, genetic variants of *DLG2* are known to be associated with altered volumes of the putamen in schizophrenia patients [15]. Disruption of multiple genes, including *DLG2*, owing to rare copy number variations (CNVs) is associated with schizophrenia [10]. On the other hand, the involvement of *DLG2* in autism spectrum disorder (ASD) has not been extensively studied. A DLG2 CNV mutation has been previously found in one ASD patient [16]. Recently, a study using large-scale whole-genome sequencing identified the recurrent deletions in the promotor region of DLG2 gene that were significantly associated with ASD, suggesting that such variations in its non-coding regulatory region may play a role in ASD [17]. In addition, studies using mice lacking exon 9 of the *Dlg2* gene have shown that DLG2 plays a role in controlling complex learning and cognitive flexibility [18] and direct social interactions [19]. Nevertheless, the mechanisms that link *DLG2*-associated mutations to autism-relevant behavioral abnormalities have remained elusive.

In the present study, using a distinct *Dlg2*-mutant mouse line lacking exon 14 of the *Dlg2* gene, we investigated whether a DLG2 deficiency might cause abnormalities in ASD-related behaviors. We found that *Dlg2* deletion leads to aberrant locomotor responses, decreased social approach, and increased repetitive behaviors and that DLG2 plays an important role in excitatory synaptic transmission in spiny projection neurons of the dorsolateral striatum. These findings provide clues regarding the mechanisms underlying DLG2-associated neurodevelopmental disorders.

## Methods

### Animals

Mice carrying a deletion of exon 14 of the *Dlg2* gene flanked by LoxP sites were designed and generated by EUCOMM and EMMA, respectively. The LacZ-Neo cassette was eliminated by crossing these mice with *protamine-Flp* mice. LacZ-Neo cassette-deleted *Dlg2*^*flox/+*^ mice were crossed with *protamine-Cre* mice, and the resulting mice were then crossed with wild-type (WT) mice to introduce the *Dlg2*^*Δ14*^ allele. Global *Dlg2*^*Δ14/Δ14*^ mice were obtained by heterozygous mating (*Dlg2*^*Δ14/+*^ x *Dlg2*^*Δ14/+*^). Mice used in this study were maintained in a C57BL/6J genetic background for more than five generations. All mice were bred and maintained at the mouse facility of Korea Advanced Institute of Science and Technology (KAIST), and all experimental procedures were approved by the Committee of Animal Research at KAIST (KA2016-28). All animals were fed ad libitum and housed under a 12-h light/dark cycle (light phase from 1:00 a.m. to 1:00 p.m.). Conventional knockout mice were genotyped by polymerase chain reaction (PCR) using the following primers: WT allele (312 bp), 5′-CCA GAA TGT AC TTC AGC ACC A -3′ (forward) and 5′-TCG TGGTATCGTTATGCGCC-3′ (reverse); and mutant allele (527 bp), 5′-GCC AAG ACT TTT AGA GAC AGC C-3′ (forward) and 5′-AAG CAG GCA ATT CAC ACC AC-3′ (reverse). Only male adult mice were used for behavioral, electrophysiological, and biochemical experiments.

### Brain lysates and western blot

The brains from 3-month-old wild-type, *Dlg2*^*Δ14/+*^, *Dlg2*^*Δ14/*Δ*14*^ mice (hereafter *Dlg2*^*+/–*^ and *Dlg2*^*–/–*^ mice, respectively) were extracted and homogenized with ice-cold homogenization buffer (0.32 M sucrose, 10 mM HEPES, pH 7.4, 2 mM EDTA, pH 8.0, 2 mM EGTA, pH 8.0, protease inhibitors, phosphatase inhibitors). Whole brain lysates were prepared by boiling with β-mercaptoethanol directly after homogenization. Total brain lysates separated in electrophoresis and transferred to a nitrocellulose membrane were incubated with primary antibodies to DLG2/PSD-93 (#1634, rabbit, as previously described [20]), DLG4/PSD-95 (Neuromab 75-028), and α-tubulin (Sigma T5168) at 4 °C overnight. Fluorescent secondary antibody signals were detected using Odyssey® Fc Dual Mode Imaging System.

### In situ hybridization

Mouse brain sections (14 μm thick) at embryonic day (E18) and postnatal days (P0, P7, P14, P21, and P56) were prepared using a cryostat (Leica CM 1950). Hybridization probe specific for mouse *Dlg2* mRNA was prepared using the following regions: nt 1116–1369 of *Dlg2* (NM_011807.3). Antisense riboprobe was generated using 35S-uridine triphosphate (UTP) and the Riboprobe System (Promega). For quantification, 15 of sampling areas (233,000 μm^2^) were randomly selected within a region of interest including the cortex, striatum, and cerebellum. The mean gray intensity of each region was measured using the ImageJ Fiji software [21]. Data were pooled from both sagittal and horizontal brain sections of two mice per each time point.

### Behavioral assays

Three-month-old male mice were used for behavioral tests. A behavioral battery was performed for all four cohorts (#1–4) with the exception of home-cage activity monitoring (cohort #1 including their heterozygous knockout littermates) and additional assays for measures of sociability (direct interaction test, cohort #4) and repetitive behavior (self-grooming test, cohorts #3 and #4). Cohorts for those additional assays were selected without prior knowledge of other behavioral phenotypes. Although the homozygous knockout mice (*Dlg2*^*-/-*^) were mainly focused to address the consequence of a DLG2 deficiency, locomotion and self-grooming behavior of the heterozygous knockout mice (*Dlg2*^+/-^) were also measured to confirm the absence of distinctive behavioral phenotypes (Table S3). Data were pooled from all cohorts that underwent the tests performed in the following order: home-cage activity monitoring, open-field test, light/dark test, elevated plus maze test, three-chamber test, direct interaction test, and self-grooming test. Subject mice were provided at least 24-h–long rest periods between tests. Animals were handled for 10 min per day for up to 5 days prior to beginning the battery of behavioral assays so as to reduce potential stress and anxiety that might be caused by an experimenter. On each day of a behavioral test, all animals were habituated to a dark room under conditions identical to those of the testing room for 30 min before starting the test. Behavioral assays were performed and analyzed by an experimenter blinded to group-identifying information. Data were analyzed using EthoVision XT 10 (Noldus), unless indicated otherwise.

### Home-cage activity monitoring

The Laboratory Animal Behavioral Observation Registration and Analysis System (LABORAS, Metris) was used for long-term monitoring of mouse movements in LABORAS cages, conditions very similar to those of home cages [22]. Mice were individually placed in a single cage within the system, and their activities were recorded for 72 consecutive hours. Locomotion and self-grooming were automatically analyzed as previously described [23, 24].

### Open-field test

Subject mice were individually placed in a white acryl box (40 × 40 × 40 cm) and video-recorded for 60 min. Light intensity was set to 120 lx. The “center” region was defined as the area of a 20 × 20 cm^2^ in the middle of the arena.

### Light/dark box test

The apparatus consists of two separate chambers (light, 21 × 29 × 20 cm, a white acryl box with no lid on top for video recording; dark, 21 × 13 × 20 cm, a black acryl box with a lid on top) as previously described [23, 25]. The light chamber was illuminated at 180 lx. The time spent in each chamber was measured and analyzed automatically.

### Elevated plus-maze test

Animals were placed in the center region of a plus-arm maze with two open (5 × 30 × 0.5 cm) and closed (5 × 30 × 30 cm, no lid on top for recording) arms. The maze was elevated to a height of 75 cm from the floor. Light intensity in the room was 150 lx. Time spent in open or closed arms and total distance moved were automatically measured.

### Self-grooming test

A subject mouse was placed in a fresh home cage without bedding, as previously described [26], and video-recorded for 30 min. The first 20 min constituted a habituation period, and self-grooming behavior was measured during the last 10 min. Self-grooming behavior was defined as a sequential activity composed of stroking and licking, as previously described [24].

### Three-chamber test

Social approach and social novelty recognition were assessed using the three-chamber test [27, 28], as described previously [23, 24]. Light intensity in the test room was 40 lx. Briefly, mice were isolated for 3 days prior to the experiment. The apparatus (60 × 40 × 20 cm) consists of three compartments; two side chambers have small containers for either a stranger mouse or a novel object. Three phases constituted one session. First (“habituation”), a subject mouse was placed in the apparatus, with the small containers in both compartments left empty, and then the mouse was allowed to freely roam in all chambers for 10 min. Second (S1-O phase), stranger mouse 1 (S1; 129/SvJae strain) was placed in the container in one side chamber, and a novel object was placed in the container in the other side chamber; S1 and O side were randomly assigned. The subject mouse was then allowed to freely move around the apparatus for 10 min. Third (S1-S2), social preference towards a new stranger (S2; 129/SvJae strain) over a “familiar” mouse (S1) was assessed by replacing the object with S2 and recording exploration of targets by the mouse for an additional 10 min. All stranger mice were age-matched with subjects.

### Dyadic social interaction

A gray Plexiglas box (30 × 30 × 30 cm) was used to measure social interaction between mouse pairs, as previously described [24, 25]. Light intensity in the testing chamber was 40 lx. Briefly, on day 1, each mouse was habituated to the testing conditions by allowing it to freely move around the box for 20 min. On day 2, a subject mouse was paired with an unfamiliar mouse (age-matched, male, 129/SvJae strain), and the mouse pairs were simultaneously placed in the testing box at diagonally opposite corners. Mouse behaviors were video-recorded for 5 min. The time spent in direct social contacts, including nose-to-nose contact, nose-to-tail contact, allo-grooming, and other body contacts, was measured as previously described [27] and analyzed by an experimenter blinded to group-identifying information.

### Electrophysiology

Acute coronal brain slices for the dorsolateral striatum were obtained following the protective recovery method [29]. In brief, mice at 2 months were anesthetized with an intraperitoneal injection of a ketamine-xylazine cocktail, followed by transcardial perfusion of a protective buffer (NMDG aCSF) at room temperature consisting of, in mM: 100 NMDG, 12 NAC, 30 NaHCO_3_, 20 HEPES, 25 glucose, 2 thiourea, 5 Na-ascorbate, 3 Na-pyruvate, 2.5 KCl, 1.25 NaH_2_PO_4_, 0.5 CaCl_2_, and 10 MgSO_4_. Mouse brains were extracted and sectioned (300 μm) in NMDG aCSF buffer at room temperature bubbled with 95% O_2_ and 5% CO_2_ gases using Leica VT 1200. And then, the resulting brain slices transferred to a 32 °C holding chamber containing NMDG aCSF for 11 min. After the incubation, the slices were transferred and recovered over 1 h in a chamber at ambient room temperature containing a recovery buffer consisting of, in mM: 92 NaCl2, 12 NAC, 30 NaHCO_3_, 20 HEPES, 25 glucose, 2 thiourea, 5 Na-ascorbate, 3 Na-pyruvate, 2.5 KCl, 1.25 NaH_2_PO_4_, 2.5 CaCl_2_, and 1.3 MgCl_2_ oxygenated with 95% O_2_ and 5% CO_2_ gases. During all recordings, brain slices were maintained in a submerge-type recording chamber perfused with 27.5 – 28.5 °C aCSF (2 ml min^−1^) (in mM: 124 NaCl, 25 NaHCO_3_, 10 glucose, 2.5 KCl, 1 NaH_2_PO_4_, 2.5 CaCl_2_, and 1.3 MgSO_4_ oxygenated with 95% O_2_ and 5% CO_2_ gases). Recording glass pipettes from borosilicate glass capillaries (Harvard Apparatus) were pulled using an electrode puller (Narishige). All electric responses were amplified and filtered at 2 kHz (Multiclamp 700B, Molecular Devices) and then digitized at 10 kHz (Digidata 1550, Molecular Devices). For whole-cell patch recordings in dorsolateral striatum, a recording pipette (2.5–3.5 MΩ) was filled with the internal solution (in mM: 100 CsMeSO_4_, 10 TEA-Cl, 8 NaCl, 10 HEPES, 5 QX-314-Cl, 2 Mg-ATP, 0.3 Na-GTP, and 10 EGTA with pH 7.25, 295 mOsm for sEPSCs; 115 CsCl_2_, 10 EGTA, 8 NaCl, 10 TEACl, 10 HEPES, 4 Mg-ATP, 0.3 Na-GTP, and 5 QX-314 with pH 7.35, 295 mOsm for sIPSC). To measure sEPSCs, and sIPSCs, dorsolateral MSN neurons were voltage-clamped at − 70 mV. For sEPSCs and sIPSCs, picrotoxin (60 μM) and NBQX (10 μM) + APV (50 μM) without TTX were added, respectively. Responses were recorded for 2 min after maintaining stable baseline for 5 min.

### Statistical analysis

Statistical analyses were performed using GraphPad Prism 7. For analysis of western blotting data, one-way analysis of variance (ANOVA) was used for assessing DLG4 expression. Data from LABORAS home-cage activity monitoring and open-field tests were analyzed by repeated measures of ANOVAs for the effects of genotype and genotype × time interactions. Post hoc analyses were performed using Sidak’s test as warranted by significant genotype × time interactions. Other data were analyzed for two-group comparisons using either Student’s *t* test for normally distributed data, as determined by the D’Agostino and Pearson test, or by non-parametric Mann-Whitney test for non-normally distributed data. Multiple group comparisons were performed for the data of in situ hybridization and home-cage activity measures with the heterozygous knockout mice using one-way ANOVA with post hoc Sidak’s test for normally distributed data or Kruskal-Wallis test with Dunn’s multiple comparisons if the normality of data was not warranted. All statistical details, including information on sample size, descriptive statistics, normality test results, and *t*, *F*, *U*, *W*, or *Z* values, are summarized in Tables S1 and S2. Differences were considered significant at *p* values < 0.05. Results are presented as means ± SE.

## Results

### A DLG2 deficiency does not affect DLG4/PSD-95 protein expression

**Fig. 1:**
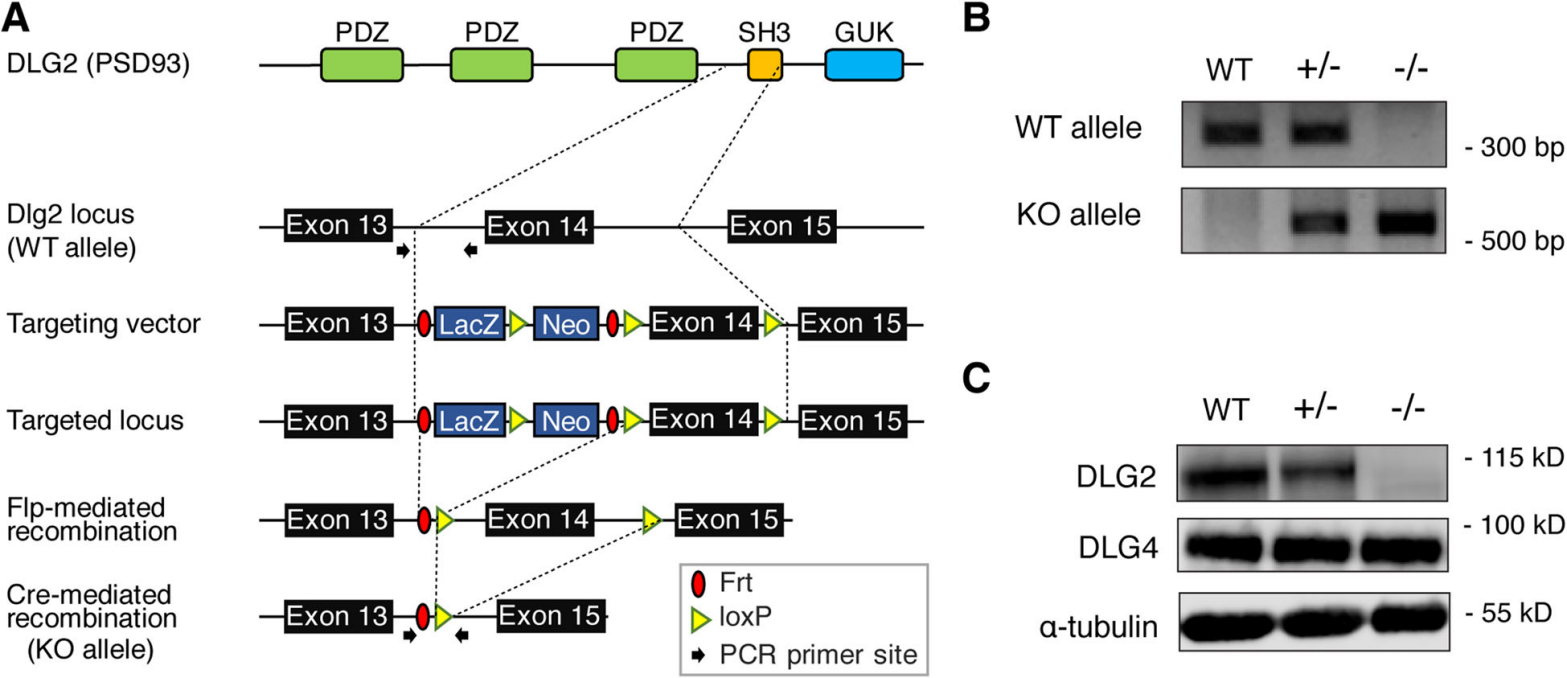
Generation and characterization of DLG2 mutant mice. **a***Dlg2* knockout (KO) strategy. **b** PCR genotyping of *Dlg2*^*+/–*^ and *Dlg2*^*–/–*^ mice. **c** DLG2 protein levels in whole brain lysates of *Dlg2*^*+/–*^ and *Dlg2*^*–/–*^ mice, determined by immunoblot analysis. There was no significant change (see text for details of quantitative data) in the expression levels of DLG4/PSD-95 protein in whole brain lysates of *Dlg2*^*+/–*^ or *Dlg2*^*–/–*^ mice

To investigate DLG2 functions in mice, we deleted exon 14 of the *Dlg2* gene by crossing *Dlg2*^*fl/fl*^ mice with *protamine-Cre* mice and crossbreeding the resulting *Dlg2*^*+/–*^ mice (Fig. 1a). The genotypes of *Dlg2*^*+/–*^ and *Dlg2*^*–/–*^ mice were confirmed by PCR (Fig. 1b). Both DLG2/PSD-93 and DLG4/PSD-95 are members of the MAGUK family that play important roles as scaffolding proteins at excitatory synapses. It has been previously reported that DLG4/PSD-95–deficient mice have increased levels of DLG2 expression, implying potential compensatory responses [19]. After first confirming knockout of DLG2 protein upon deletion of exon 14 of the *Dlg2* gene (Fig. 1c), we investigated possible issues associated with functional redundancy [3] by assessing DLG4 expression. DLG4 protein levels were unchanged in *Dlg2*^*+/–*^ and *Dlg2*^*–/–*^ mice compared with WT mice (1.08 ± 0.27-fold and 1.11 ± 0.20-fold for *Dlg2*^*+/–*^ and *Dlg2*^*–/–*^, respectively, relative to WT levels; one-way ANOVA, *F*_(2, 9)_ = 0.07, *p* = 0.93).

### *Dlg2*^*–/–*^ mice show aberrant locomotor responses to novelty

**Fig. 2:**
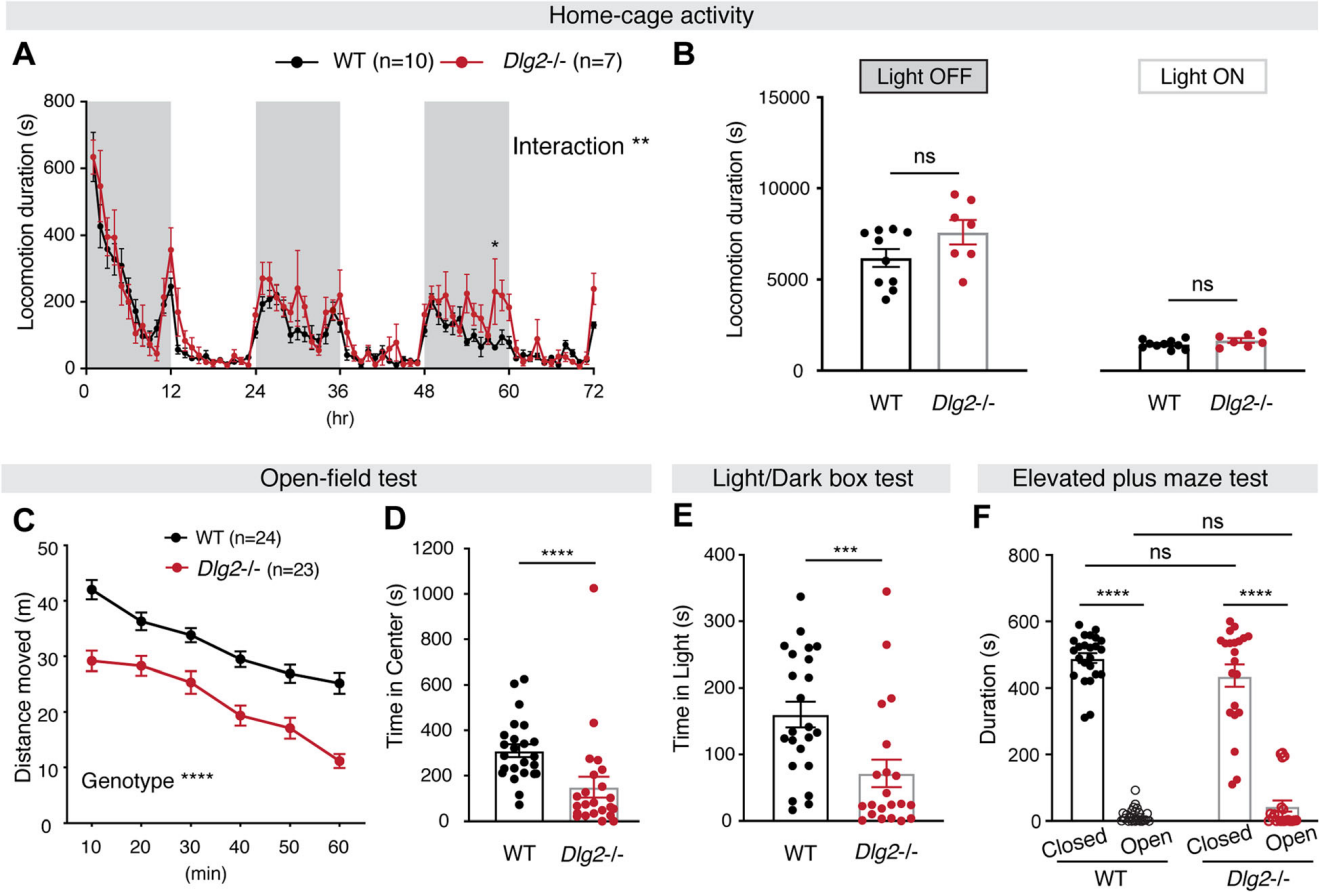
*Dlg2*^*–/–*^ mice display novelty-induced hypoactivity and moderately increased anxiety-like behavior. **a**, **b** In the home-cage–like setting of the LABORAS system, there was no difference in overall locomotion between genotypes, with no main effect of genotype. However, there was a significant interaction effect of time × genotype (“Interaction”), revealing heightened locomotion in *Dlg2*^*–/–*^ mice compared to WT mice at 58 h. WT, *n* = 10; *Dlg2*^*–/–*^, *n* = 7; repeated measures of two-way ANOVA. **c** In the open-field test, *Dlg2*^*–/–*^ mice showed significantly decreased locomotion compared to WT animals throughout the test duration. Repeated measures two-way ANOVA. In open-field arena and light/dark box tests, *Dlg2*^*–/–*^ mice spent significantly less time in the center region of the open-field arena (**d**) and in the lighted box (**e**) compared with WT mice. Mann-Whitney test. **f** In the elevated plus-maze, there was no difference between genotypes in the time spent in open versus closed arms. WT, *n* = 24; *Dlg2*^*–/–*^, *n* = 23 for **c**–**f**. *p < 0.05; **p < 0.01; ***p < 0.001; ****p < 0.0001; ns, not significant; two-way ANOVA

Previous studies have reported decreased open-field locomotion of DLG2- and DLG4-deficient mice [19, 30]. To investigate whether this abnormal-locomotion phenotype is replicated in *Dlg2*^*–/–*^ mice lacking exon 14, we tested locomotor traits in two different settings: home cage and open-field arena (Fig. 2). Monitoring of animals for 72 h in LABORAS cages (home-cage environment) revealed no difference in the total amount of locomotion (Fig. 2a, b; see Table S3 for measures in *Dlg2*^*+/–*^ mice), consistent with previous findings from the aforementioned studies using mice lacking *Dlg2* exon 9 [19]. In contrast, we found that *Dlg2*^*–/–*^ mice showed significantly less exploratory behavior in the open-field test arena than WT animals (Fig. 2c). These results suggest that mutations in the *Dlg2* gene cause aberrant motor responses in a novel environment, such as a brightly lit open arena, but not in a home-cage setting.

With the decreased exploration, *Dlg2*^*–/–*^ mice spent less time in the center region of the open-field arena (Fig. 2d). Furthermore, *Dlg2*^*–/–*^ mice showed a significantly decreased duration of exploring in the light chamber of the light/dark box test (Fig. 2e), a behavioral test commonly used to measure the level of anxiety in rodents [31]. However, there was no difference in exploration between *Dlg2*^*–/–*^ and WT mice in the elevated plus-maze test (Fig. 2f). Collectively, given that such behavioral measures of anxiety in rodents greatly depend on the locomotion of a subject [31], these results with normal locomotion in the home-cage setting suggest that *Dlg2*^*–/–*^ mice exhibit context-dependent aberrant locomotor responses to novelty.

### Social approach and interaction are reduced in *Dlg2*^–/–^ mice

**Fig. 3:**
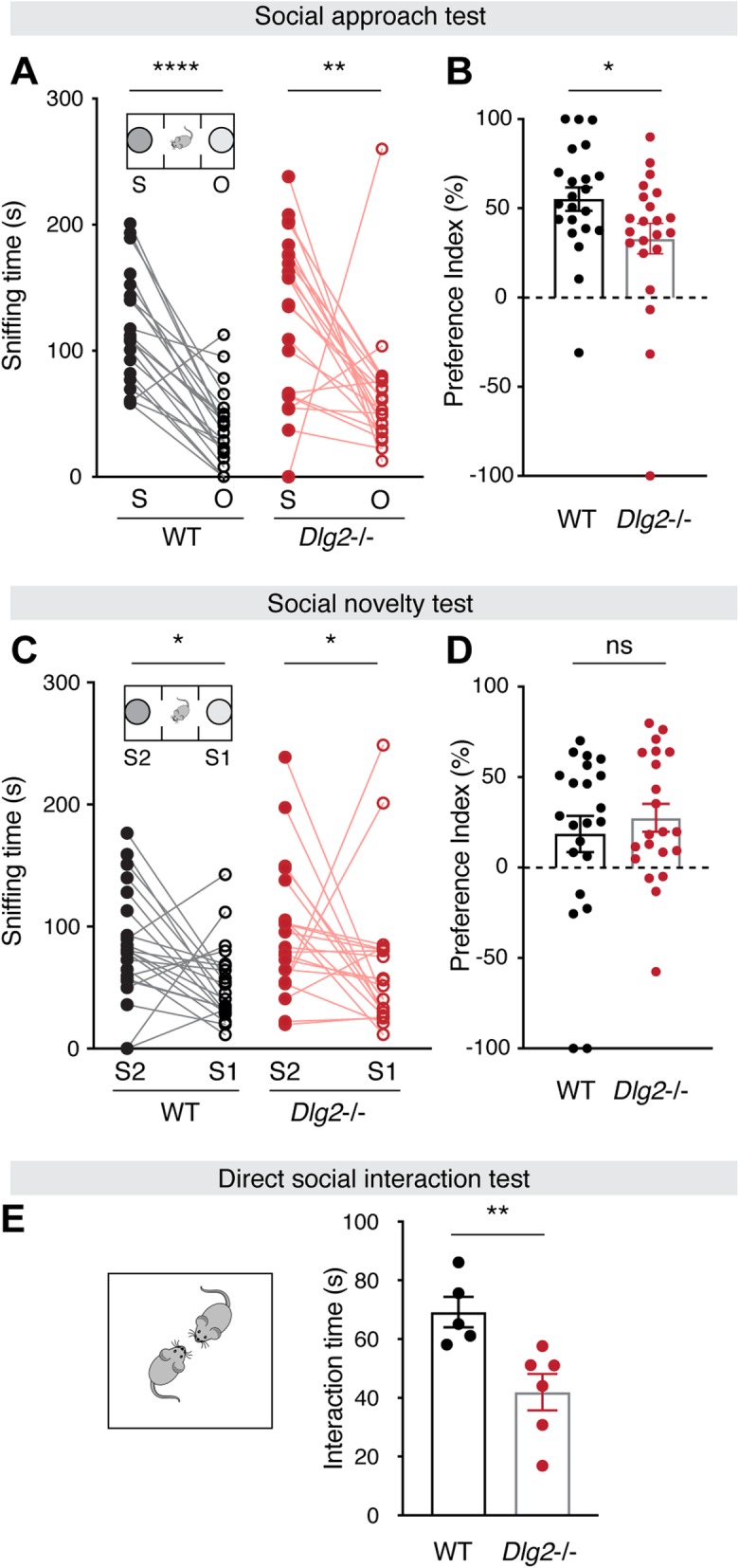
*Dlg2*^*–/–*^ mice display decreased social approach but normal social novelty recognition. **a** Both WT and *Dlg2*^*–/–*^ mice spent more time sniffing an age-matched stranger mouse (S) than a novel object (O). **b** The preference index (S-O) of *Dlg2*^*–/–*^ mice, which was calculated for group comparisons as %[(time spent in sniffing S − time spent in O)/total time spent (S + O) × 100], was significantly decreased compared to that of WT. *n* = 22 mice per genotype. **c**, **d** There was no difference between groups in the social novelty test, as shown by the time spent sniffing a novel stranger mouse (S2) and a familiar mouse (S1). The preference index for the S1-S2 phase was not significantly different between groups. *n* = 22 mice per genotype. **e***Dlg2*^*–/–*^ mice tended to spend less time interacting with an age-matched stranger (WT, 129/SvJae) than with WT mice in the test arena, where mice were allowed to freely explore and interact each other. WT, *n* = 5; *Dlg2*^*–/–*^, *n* = 6. Wilcoxon matched-pairs signed rank tests were used for **a** and **c**. Mann-Whitney tests were used for **b**, **d**, and **e**. *p < 0.05; **p < 0.01; ****p < 0.0001; ns, not significant

Given that aberrant sociability is one of the key ASD-related phenotypes in mouse models of ASD, we tested whether *Dlg2* deletion affects social behaviors. These tests sought to assess three aspects: social approach, social novelty recognition, and direct interaction. In the three-chamber test, used to assess social approach, *Dlg2*^*–/–*^ mice spent a significantly increased amount of time sniffing a social stimulus (“S”) over a novel object, implying a preference toward a social stimulus over a novel object (‘O’; Fig. 3a). However, the preference index, S-O, calculated as %[(time spent sniffing S − time spent in O)/total time spent (S + O)], used for group comparisons of the extent of the preference, was significantly decreased (Fig. 3b), as previously described [25, 32]. Note that the decrease was found to be statistically significant, either with or without an outlier (see Table S1 for details). On the other hand, there was no group difference in approach preference toward social novelty (S1-S2 preference index, %[(time spent sniffing S2 − time spent in S1)/total time spent (S2 + S1)]), indicating that *Dlg2*^*–/–*^ mice did not have overt abnormalities in recognizing a novel social stimulus over a familiar one or approaching the social novelty (Fig. 3c, d). Additionally, when allowed to freely engage in direct interaction with a wild-type novel mouse of a different strain, the mutant mice tended to spend less time in direct interaction than WT animals (Fig. 3e). These findings suggest that a DLG2 deficiency alters the extent of social approach and direct social interaction, without significantly affecting social novelty recognition.

### A DLG2 deficiency leads to increased self-grooming behavior

**Fig. 4:**
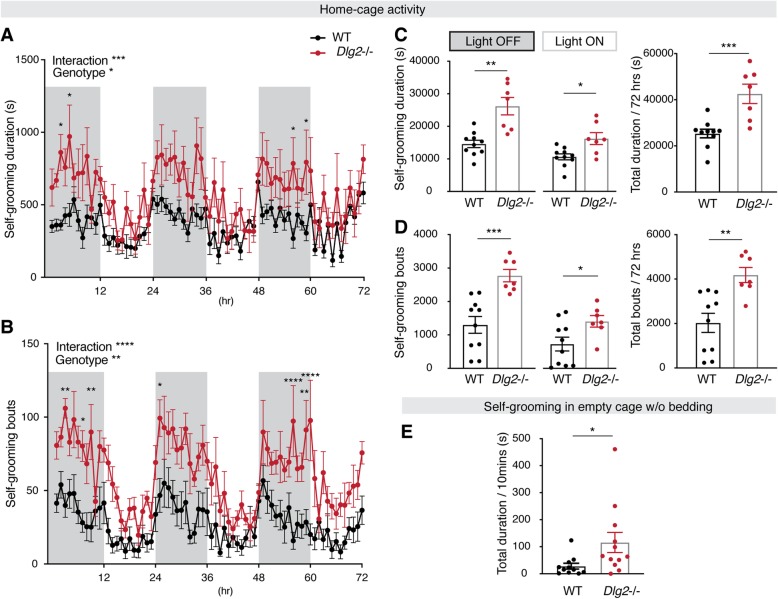
*Dlg2*^*–/–*^ mice display increased self-grooming behavior. Monitoring of behavior in LABORAS cages (home-cage activity) for 72 h showed that both the duration (**a**) and number (**b**) of bouts of self-grooming were significantly increased in *Dlg2*^*–/–*^ mice compared with WT animals. **c**, **d** Increased duration and number of bouts of self-grooming in *Dlg2*^*–/–*^ mice were found regardless of light on or off conditions, resulting in significant increases in total duration and number of bouts of self-grooming. WT, *n* = 10; *Dlg2*^*–/–*^, *n* = 7. **e** Measurement of self-grooming behavior in an empty cage without bedding for 10 min showed that *Dlg2*^*–/–*^ mice also exhibited a significantly increased duration of self-grooming in this different setting. WT, *n* = 11; *Dlg2*^*–/–*^, *n* = 12. Repeated measures of ANOVA was used for **a** and **b**, with post hoc Sidak’s multiple comparisons test applied under conditions where there was a significant effect of interaction (noted in **a** and **b**). Mann-Whitney tests were used for **c**–**e**. *p < 0.05; **p < 0.01; ***p < 0.001; ****p < 0.0001

Increased self-grooming in rodents is a commonly assessed indicator of repetitive behavior that is believed to model behavioral perseveration in ASD [26]. Indeed, various studies have shown that increased self-grooming is one of the key behavioral traits in animal models of ASD [33–35]. To determine whether DLG2 is associated with repetitive behavior, we measured the level of self-grooming in home cages with a long-term monitoring system (LABORAS system) or in an empty cage without bedding (test setting; see Methods for details). Self-grooming was significantly increased in *Dlg2*^*–/–*^ mice compared with WT mice (Fig. 4). Quantitative increases in self-grooming included both cumulative duration (Fig. 4a, c) and the number of bouts (Fig. 4b, d). Note that the heterozygous knockout (*Dlg2*^*+/-*^) mice showed moderate increases in the self-grooming duration and no differences in the bouts compared to WT mice (Table S3). While locomotor activity in *Dlg2*^*–/–*^ mice varied with the type of setting (Fig. 1), self-grooming was consistently increased in either setting (Fig. 4e). Notably, increases in self-grooming behavior measured in the home cage persisted regardless of lights-on or -off conditions (Fig. 4c, d), implying that the mutant mice have a sleep disturbance, as is commonly observed in ASD patients [36, 37]. These results suggest that a DLG2 deficiency is associated with increased repetitive behavior.

### Excitatory synaptic transmission is decreased in dorsal striatal spiny projection neurons of *Dlg2*^*–/–*^ mice

**Fig. 5:**
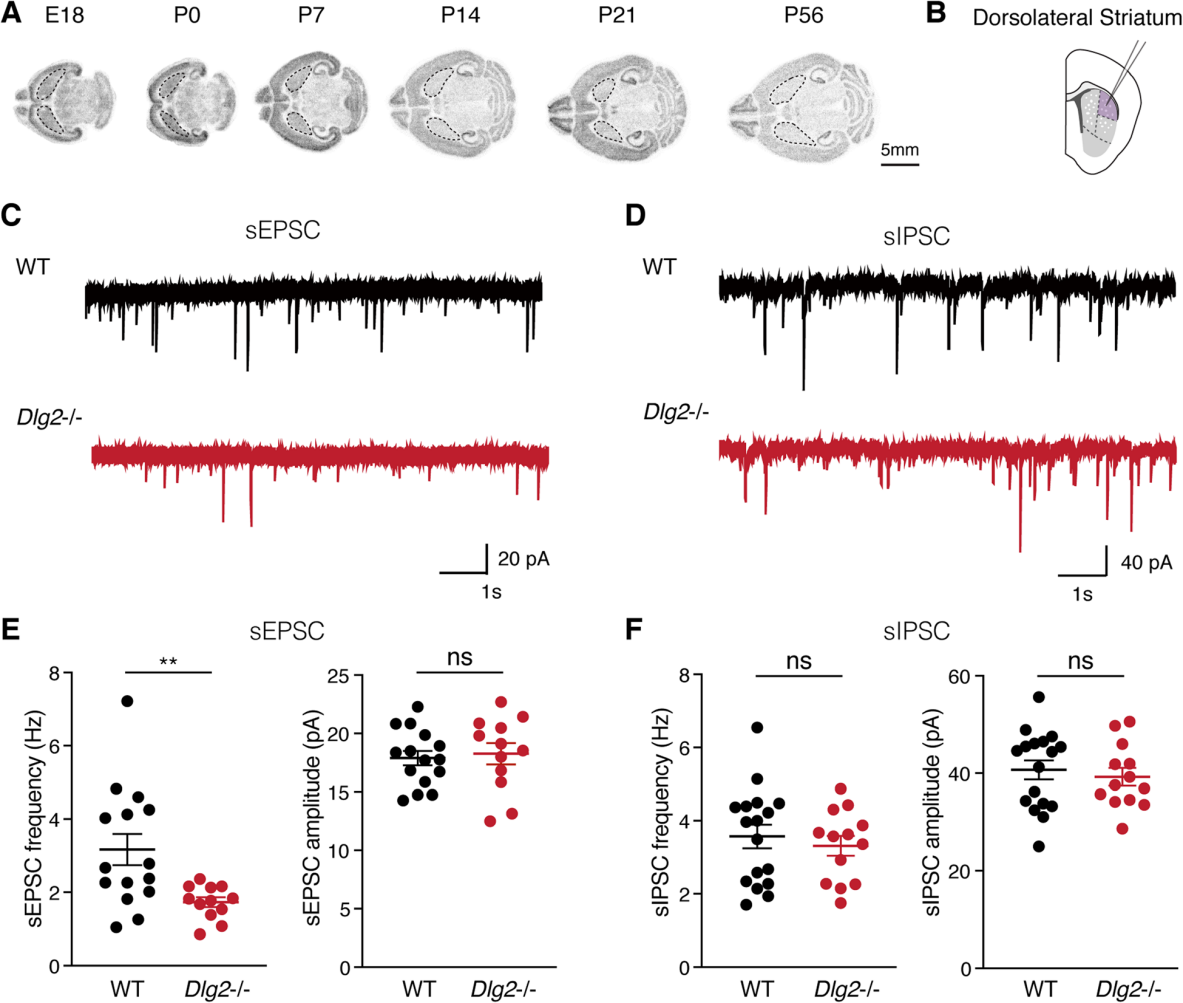
Excitatory synaptic transmission is decreased in dorsal striatal SPNs of *Dlg2*^*–/–*^ mice. **a***Dlg2* mRNA levels in the mouse brain during development, revealed by in situ hybridization of horizontal sections. Caudate putamen (striatum) regions are delineated by dotted lines on each section. *Dlg2* mRNA expression was increased during early development until P7 (see Fig. S1). **b** Illustration of the dorsolateral striatum region where electrophysiological recordings were made. **c**, **d** Representative electrophysiological traces showing spontaneous excitatory postsynaptic currents (sEPSCs, **c**) and spontaneous inhibitory postsynaptic currents (sIPSCs, **d**) recorded in spiny projection neurons (SPNs) of the dorsolateral striatum of WT versus *Dlg2*^*–/–*^ mice. **e** The frequency, but not the amplitude, of sEPSCs was significantly decreased in SPNs of *Dlg2*^*–/–*^ mice compared with that in WT mice (*n* = 15 neurons from three WT mice and *n* = 12 neurons from three *Dlg2*^*–/–*^ mice). **f** Similar frequency and amplitude of sIPSCs in the dorsolateral striatum of *Dlg2*^*–/–*^ mice were found. (*n* = 17 neurons from three WT and *n* = 13 neurons from three *Dlg2*^*–/–*^ mice). **p < 0.01; ns, not significant. Student’s *t* tests were used for **e** and **f**

Increased repetitive behaviors, deficits in social interaction, and aberrant locomotor responses are known to be linked to synaptic dysfunction in the dorsal striatum [38–40]. Since we found that *Dlg2*^*–/–*^ mice exhibit increased self-grooming, decreased locomotion in a bright arena, and decreased sociability, we hypothesized that a DLG2 deficiency affects synaptic function in the dorsal striatum. We first confirmed expression of DLG2 in the dorsal striatum of the mouse brain. It should be noted that, during early development, DLG2 is highly expressed in the putamen of the human brain [41], the region homologous to the dorsal striatum in mice. In situ hybridization analyses revealed that *Dlg2* mRNA levels were elevated up to postnatal day 7 (P7) in the WT mouse brain (Fig. 5a, also in Fig.S1), implying prominent *Dlg2* expression at early stages of brain development, similar to the case in the human brain.

We then investigated the physiological ramifications of a DLG2 deficiency in the dorsolateral striatum of adult mice (Fig. 5b). Spontaneous excitatory postsynaptic currents (sEPSCs) and spontaneous inhibitory postsynaptic currents (sIPSCs) of spiny projection neurons (SPNs), the output cell type in the striatum, were measured in the presence of network activity that was enabled by excluding tetrodotoxin, which blocks action potential firing, from brain slice preparations used for recording. The frequency, but not amplitude, of sEPSCs was significantly decreased in dorsolateral striatal SPNs of *Dlg2*^*–/–*^ mice compared with those from WT animals (Fig. 5c, e). In contrast, neither the frequency nor amplitude of inhibitory synaptic transmission in the dorsolateral striatum was affected by a DLG2 deficiency (Fig. 5d, f). These results indicate that genetic deletion of *Dlg2* exon 14 leads to reduced excitatory synaptic inputs onto SPNs, without significantly affecting the AMPA receptor function in the dorsolateral striatum of mice.

## Discussion

Previous studies have highlighted a close association between mutations in *Dlg2* and schizophrenia [9–13]. Here, we present results indicating that a DLG2 deficiency induces autism-related behavioral phenotypes, including aberrant locomotor responses to novelty, decreased social approach, and significantly increased repetitive behavior. Furthermore, *Dlg2* knockout in mice resulted in reduced excitatory synaptic inputs onto SPNs, the output cell type of the striatum. Given that the striatum is known to be related to locomotor, social, and repetitive behaviors [26, 39, 40], we suggest that DLG2 gene disruption can lead to dysfunctional synaptic inputs in the striatum and relevant behavioral abnormalities.

Aberrant striatal circuits have been implicated in neurodevelopmental disorders, including schizophrenia [42, 43], obsessive-compulsive disorder [38, 44], and autism spectrum disorders [45–47]. Interestingly, emerging evidence supports the involvement of DLG2 in striatal development in the human brain. During mid-fetal development in humans, DLG2 is highly expressed in the striatum [41]. Mutations in DLG2 alter the volume of the putamen, a part of the dorsal striatum in humans [48]. In patients with schizophrenia, genetic variants of DLG2 are linked to aberrant structural features of the putamen [15]. Based on the notable abundance of *Dlg2* mRNA expression in the mouse striatum at early developmental stages (from embryonic day 18 up to the first week of postnatal development, Fig. 5a and Fig. S1), it seems reasonable to postulate that a DLG2 deficiency influences striatal circuit development. Notably, the intrinsic excitability of SPNs is enhanced until the first week of postnatal development in rodents and starts decreasing from P10 [49, 50]. Furthermore, SPN maturation is tightly regulated by inward rectifier potassium (K_ir_) channels [50, 51]. Since DLG2 interacts directly with K_ir_ channels [3, 52], it may play a role in shaping the functional maturation of SPNs during a critical period of striatal circuit development. Further investigation of the mechanisms by which *Dlg2* mutations affect the activity of K_ir_ channels with respect to regulation of the excitability of SPNs and striatal maturation is warranted.

Self-grooming in rodents involves a complex patterned sequence of motor activities. Increased self-grooming behavior in rodents is thought to represent pathological repetitive behavior (i.e., behavioral perseveration), which is one of the core symptoms of ASD [26]. Accordingly, animal models that display increased self-grooming behavior have been used to investigate the mechanisms underlying ASD symptoms [53–55]. It is well known that repetitive self-grooming behavior in rodents is strongly associated with dysfunction of the striatum [38, 40]. Aberrant connectivity related to the striatum is also thought to be a major culprit in the repetitive behavior of humans with ASD [45–47]. It should be noted that *Dlg2* knockout induced a significant increase in self-grooming behavior during home-cage activity monitoring, even in “light-on” periods (Fig. 4), when rodents normally sleep. This implies that these mutant mice might have disrupted sleep patterns. Sleep problems commonly occur not only in patients with ASD [36, 37], but also in those with movement disorders such as Parkinson’s disease [56, 57]. Although control of sleep is intricately regulated by numerous brain regions and their complex connectivity [58], emerging evidence supports the conclusion that striatal circuits are highly associated with sleep. For example, the effect of sleep on motor sequence learning is related to the neural activity of the striatum [59]. Firing patterns of striatal SPNs changes in accordance with the sleep-wake cycle [60]. Notably, genes relevant to sleep control are dysregulated in the striatum of several mice models of Parkinson’s disease [61]. Given that NMDA receptor activity regulates sleep rhythm [62] and DLG2 directly interacts with NMDA receptors [3], it seems possible that DLG2 is involved in the regulation of sleep. Nevertheless, what type of sleep problems *Dlg2*^-/-^ mice have, how the reduced excitatory synaptic transmission of striatal SPNs affects sleep disturbance, or even whether the sleep problems result in synaptic dysfunction of SPNs and repetitive behavior remain unclear.

Since the initial cloning and characterization of DLG2 under the name Chapsyn-110 [3], several studies have used DLG2-deficient mice in which exon 9 of the *Dlg2* gene is deleted (*Dlg2*^ΔE9/ΔE9^) to investigate the role of DLG2 in synaptic transmission in the cerebellum and hippocampus as well as in the control of several behavioral traits [6, 7, 18, 19]. Here, we used mice with a targeted deletion of exon 14 (Fig. 1). Similar to findings obtained using *Dlg2*^ΔE9/ΔE9^ mice, we found aberrations in locomotion that depended on the test setting (home-cage vs. open-field arena) in these mutant mice. However, we found mild decreases in the sociability of these mutant mice, as measured by both three-chamber and direct social interaction tests, whereas Winkler and colleagues [19] reported that *Dlg2*^ΔE9/ΔE9^ mice exhibit hypersociability, as measured by the direct interaction test. While it is conceivable that deletion of different exons generates mixed results in sociability, as has been found among different *Shank3* models of ASD [63, 64], differences in testing conditions for direct social interactions might contribute to the discrepancy. For instance, the strangers used in the present study were naïve wild-type mice of a different strain, 129/SvJae, whereas the aforementioned study used mice of the same strain and genotype as stranger mice [19]. It should be noted that the level of sociability substantially varies with mouse strain [28, 65], and the strain of a stranger mouse can influence social behaviors of a subject mouse [66]. Furthermore, the light intensity in the testing room was much lower in the present study (40 lx) than in the previous study (130 lx [19]; note that we used 120 lx for open-field tests). We designed the direct interaction test with the goal of creating an encounter with a stranger with heightened novelty (i.e., a strain with different colored fur) and a consistent level of sociability for direct comparisons between groups (i.e., a wild-type mouse as an interactor for both genotypes), while ruling out other potentially stressful factors, such as bright light. Given that *Dlg2*-knockout mice in the present study displayed context-dependent abnormalities in locomotion and anxiety-like behaviors (Fig. 2), differences in the extent of strangers’ novelty and light intensity during tests might lead to discrepant results.

Interestingly, DLG2-deficient mice consistently exhibited a normal level of locomotion in a home-cage setting but showed significantly reduced locomotor activity in open and bright arenas. This reduced locomotor activity was accompanied by decreased time spent exploring the center region of the open-field arena (120 lx) and the lighted chamber (180 lx) in the light/dark box test, corroborating the strong tendency of *Dlg2*^-/-^ mice to avoid novel, open, and bright areas. These results can be partly explained by the role of the striatum in locomotor responses to novel or aversive stimuli. In a recent study, mice lacking the oxytocin receptor, a well-known animal model that displays autistic-like behaviors exhibited impaired approach to novelty in association with altered expression of excitatory synaptic markers in the dorsolateral striatum, but not the hippocampus [67]. Low novelty-seeking and high harm-avoidance behaviors are tightly associated with altered striatal connectivity to the limbic and frontal cortical regions [68]. Furthermore, a subset of dopaminergic neurons projecting in the striatum is involved in reinforcing the avoidance of threatening stimuli [69]. Given that excitatory synaptic inputs onto SPNs in the dorsolateral striatum play a crucial role in initial processing for action selection [45], the presence of such a strong form of avoidance in the mutant mice would seem to substantiate dysfunction of striatal circuitry.

The decreased frequency but not altered amplitude of sEPSCs recorded in striatal SPNs of *Dlg2*^-/-^ mice indicates that a DLG2 deficiency resulted in reduced excitatory synaptic inputs without much affecting the AMPA receptor function in SPNs. This brings up important questions about the causation of it. Striatal SPNs are receiving excitatory synaptic inputs mainly from the cortex and thalamus [45]. It is well known that the corticostriatal projection plays an important role in the regulation of repetitive behavior, as previously found in the studies using animal models of obsessive-compulsive disorder [70] and ASD [47, 71]. Given that the expression levels of *Dlg2* mRNA are abundant not only in the striatum but also the cortex during early development (Fig. 5a and Fig. S1), dysfunction of the corticostriatal circuitry might account for the decreased synaptic inputs onto SPNs in *Dlg2*^-/-^ mice. On the other hand, emerging evidence has corroborated the importance of the thalamic projections onto SPNs in the dorsal striatum in controlling repetitive and habitual behaviors involving a motor sequence [72, 73]. Therefore, we cannot rule out the possibility that deficits in the thalamostriatal circuitry might in part contribute to the reduced excitatory synaptic inputs onto SPNs and the enhanced repetitive behavior of the mutant mice. In addition to the afferent projections from distal regions, changes in striatal local circuitry might be one of the culprits as well, given the essential role of the complex interactions among various types of neurons in the regulation of synaptic transmission in SPNs [74]. For example, nicotinic acetylcholine receptors that are abundantly expressed on cholinergic synapses tightly control glutamatergic synaptic inputs onto SPNs in the dorsal striatum [75]. Based on the previous finding that DLG2 regulates the stability of cholinergic synapses [76], the decreased frequency of sEPSCs in SPNs might have resulted from altered intrastriatal circuitry via dysfunction of the cholinergic system owing to a DLG2 deficiency. Further studies are required to fully dissect the mechanisms by which DLG2 gene disruption results in the reduced excitatory synaptic inputs onto striatal SPNs and how it is related to behavioral aberration.

## Limitations

As noted above, we focused on the dorsolateral striatum primarily based on the striatum-related behavioral traits and the association of DLG2 with the putamen development in the human brain. Although this focus reflected our view of the brain region with the most plausible connection to behavioral phenotypes observed in the mutant mice, it does not rule out the possible involvement of other brain regions. It should also be noted that we have demonstrated that a DLG2 deficiency is associated with striatal synaptic aberrations and behavioral abnormalities, but this does not necessarily mean that such altered synaptic transmission in the striatum is causative. Furthermore, because the present study was designed to address the question of whether a DLG2 deficiency in mice is linked to the manifestation of ASD behavioral symptoms, we employed a homozygous knockout model. In addition to the findings of the present study, future studies using animal models of specific mutations or dysregulation of DLG2 identified in patients will provide further translationally relevant evidence supporting efforts to understand the pathological mechanisms underlying ASD.

## Conclusions

In the present study, we present results indicating that genetic disruption of *Dlg*2, which has been mainly regarded as a schizophrenia-susceptibility gene, leads to abnormalities in ASD-relevant behavioral realms, such as sociability and repetitive behavior. While DLG4/PSD-95 has been extensively studied with respect to its association with neurodevelopmental disorders, the results of the present study suggest a potential role for DLG2/PSD-93 in ASD as well. Furthermore, a DLG2 deficiency resulted in reduced excitatory synaptic inputs onto SPNs in the dorsolateral striatum. While the hippocampus and cerebellum have been the main focus in terms of the role of DLG2 in synaptic transmission, the present study revealed a role for DLG2 in striatal synaptic transmission. Given that DLG2 is highly abundant in the striatum during early development in both human and mouse brains, these findings provide clues that may help elucidate the involvement of DLG2 in neurodevelopmental disorders, including ASD, potentially through its influence on striatal circuits.

## Supplementary information

**Additional file 1: Table S1**. A full list of detailed information about statistics used for behavioral experiments presented in Figs. 2, 3,4. **Table S2.** The statistics used for analyses of electrophysiological recording and *in situ* hybridization quantification. Both tables include the number of animals, variables for comparisons, statistics used, normality test results, and notes regarding identified outliers. **Table S3.** The measures of locomotion and self-grooming behavior of the heterozygous knockout (*Dlg2*^+/-^) mice. Kruskal-Wallis tests were used for multiple comparisons including the heterozygous group.

**Additional file 2: Figure S1.** (A) *In situ* hybridization of sagittal sections reveals *Dlg2* mRNA levels in the mouse brain at embryonic day 18 (E18), postnatal day 0, 7, 14, 21, and 56 (P0, P7, P14, P21, and P56, respectively). Striatum regions are delineated by dotted lines on each section. The scale bar is 5 mm. (B) The *Dlg2* mRNA expression was found to vary with developmental stages. Note that the mRNA levels are notably heightened until P7 and relatively low throughout the brain in adults. n=15 of two mice at each time point. Based on the normality of data, one-way ANOVA with Sidak’s multiple comparisons for the cortex and Kruskal-Wallis test with Dunn’s multiple comparisons for the striatum and cerebellum were used. ns, not significant, **p < 0.01, ***p<0.001, ****p<0.0001 vs. adult (P56).

## Data Availability

All data generated or analyzed during this study are included in this published article and additional files.

## Abbreviations

AMPA: α-Amino-3-hydroxy-5-methyl-4-isoxazolepropionic acid
ASD: Autism spectrum disorders
CNV: Copy number variation
DLG2: Discs large MAGUK scaffold protein 2
DLG4: Discs large MAGUK scaffold protein 4
K_ir_ channels: Inward rectifier potassium channels
KO: Knockout
mRNA: Messenger ribonucleic acid
NMDA: N-methyl-D-aspartate receptor
PSD-93: Postsynaptic density protein-93
PSD-95: Postsynaptic density protein-95
sEPSC: Spontaneous excitatory postsynaptic current
sIPSC: Spontaneous inhibitory postsynaptic current
SPN: Spiny projection neuron
WT: Wild-type
