# Supplementary figures and images for: A DLG2 deficiency in mice leads to reduced sociability and increased repetitive behavior accompanied by aberrant synaptic transmission in the dorsal striatum
Source: Mol Autism. 2020 Mar 12;11:19. doi: 10.1186/s13229-020-00324-7 (PMC7069029; doi:10.1186/s13229-020-00324-7)

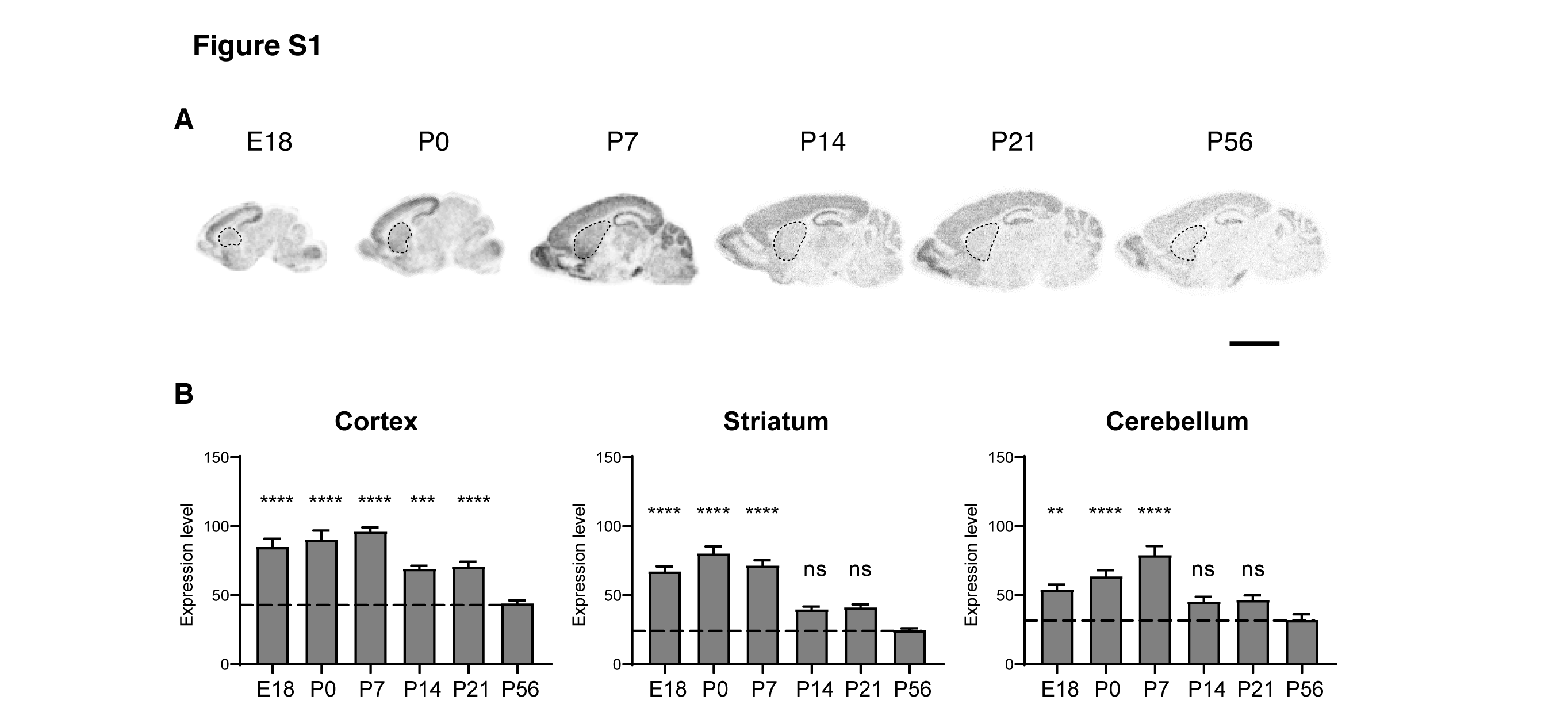

Supplement: Supplementary file 2 — Additional file 2: Figure S1. (A) In situ hybridization of sagittal sections reveals Dlg2 mRNA levels in the mouse brain at embryonic day 18 (E18), postnatal day 0, 7, 14, 21, and 56 (P0, P7, P14, P21, and P56, respectively). Striatum regions are delineated by dotted lines on each section. The scale bar is 5 mm. (B) The Dlg2 mRNA expression was found to vary with developmental stages. Note that the mRNA levels are notably heightened until P7 and relatively low throughout the brain in adults. n=15 of two mice at each time point. Based on the normality of data, one-way ANOVA with Sidak’s multiple comparisons for the cortex and Kruskal-Wallis test with Dunn’s multiple comparisons for the striatum and cerebellum were used. ns, not significant, **p < 0.01, ***p<0.001, ****p<0.0001 vs. adult (P56). [file 13229_2020_324_MOESM2_ESM.tif]
